# Neonatal T Helper 17 Responses Are Skewed Towards an Immunoregulatory Interleukin-22 Phenotype

**DOI:** 10.3389/fimmu.2021.655027

**Published:** 2021-05-03

**Authors:** Hamid R. Razzaghian, Zohreh Sharafian, Ashish A. Sharma, Guilaine K. Boyce, Kelsey Lee, Rachel Da Silva, Paul C. Orban, Rafick-Pierre Sekaly, Colin J. Ross, Pascal M. Lavoie

**Affiliations:** ^1^ BC Children’s Hospital Research Institute, University of British Columbia, Vancouver, BC, Canada; ^2^ Department of Pediatrics, Faculty of Medicine, University of British Columbia, Vancouver, BC, Canada; ^3^ Experimental Medicine Program, Department of Medicine, Faculty of Medicine, University of British Columbia, Vancouver, BC, Canada; ^4^ Department of Pathology & Laboratory Medicine, School of Medicine, Emory University, Atlanta, GA, United States; ^5^ Department of Surgery, Faculty of Medicine, University of British Columbia, Vancouver, BC, Canada; ^6^ Faculty of Pharmaceutical Sciences, Faculty of Medicine, University of British Columbia, Vancouver, BC, Canada

**Keywords:** Stat3, gene regulation, T helper (Th) 17 cells, TGF-β signaling, neonatal, T cells

## Abstract

Newborns are frequently affected by mucocutaneous candidiasis. Th17 cells essentially limit mucosal invasion by commensal *Candida* spp. Here, we sought to understand the molecular basis for the developmental lack of Th17 cell responses in circulating blood neonatal T cells. Naive cord blood CD4 T cells stimulated in Th17-differentiating conditions inherently produced high levels of the interleukin-22 immunoregulatory cytokine, particularly in the presence of neonatal antigen-presenting cells. A genome-wide transcriptome analysis comparing neonatal and adult naïve CD4 T cells *ex vivo* revealed major developmental differences in gene networks regulating Small Drosophila Mothers Against Decapentaplegic (SMAD) and Signal Transducer and Activator of Transcription 3 (STAT3) signaling. These changes were functionally validated by experiments showing that the requirement for TGF-β in human Th17 cell differentiation is age-dependent. Moreover, STAT3 activity was profoundly diminished while overexpression of the *STAT3* gene restored Th17 cell differentiation capacity in neonatal T cells. These data reveal that Th17 cell responses are developmentally regulated at the gene expression level in human neonates. These developmental changes may protect newborns against pathological Th17 cell responses, at the same time increasing their susceptibility to mucocutaneous candidiasis.

## Introduction

T helper 17 (Th17) cells are essential to limit *Candida* invasion at the skin and mucosal surfaces; this is evidenced by observations of loss-of-function genetic mutations in IL-17, and related receptor gene pathways, resulting in severe, recurrent mucocutaneous Candidiasis (MC) (1). Neonates are also susceptible to MC, which frequently manifests as oral thrush and diaper rashes, but the underlying mechanisms are not well understood (2). In full-term neonates, *Candida* species trigger robust *in vitro* innate immune responses (3). Neonatal T cells are also able to produce strong IL-17 responses in lymph nodes and the gut (4). However, according to other studies, peripheral blood naïve T cells produce only weak Th17 responses (5, 6), and IL-17-producing cells in the blood circulation are restricted to a subset of CD45RO^+^CCR7^-^CD25^low^ CCR6^+^ effector memory (Tem) cells (7). These observations raise important questions about the mechanisms involved in these developmental changes in humans, and how they impact the ability of neonates to respond they to pathogens such as *Candida* species.

Functionally, neonatal T cells are biased towards Th2 or T regulatory cell differentiation (8). Earlier studies show that fetal CD4 T cells are transcriptionally distinct from their adult counterparts (9). To our knowledge there has been little studies directly comparing gene expression profiles in term neonatal and adult naïve T cells, at the genome-wide level. In mice, IL-6 and Transforming Growth Factor beta (TGF-β) are sufficient for Th17 cell differentiation (10). In humans, Th17 cells differentiation occurs in the presence of IL-1β and IL-23 alone (11). In light of these findings, Acosta-Rodriguez et al. proposed that TGF-β is not essential during human Th17 cell differentiation (12). As Zhang has pointed out (13), experiments showing that TGF-β is essential during human Th17 cell differentiation used cord blood, whereas experiments supporting that TGF-β is not required, used adult blood. This raises the possibility that previous observations reflect age-dependent TGF-β requirements during the Th17 differentiation of human naïve CD4 T cells.

In CD4 T cells, binding of IL-6 and IL-23 through their receptors results in phosphorylation of the Signal Transducer and Activator of Transcription 3 (STAT3), which in turn leads to activation of *RORC*, the gene encoding the master Th17 transcriptional regulator RAR-related Orphan Receptor gamma (RORγt) (14). Basic Leucine Zipper ATF-Like Transcription Factor (BATF) and Interferon Regulatory Factor 4 (IRF4) cooperate to increase accessibility of chromatin to STAT3 and RORγt leading to Th17 cell differentiation (10, 15). Compared to adults, RORC is poorly expressed in naive neonatal CD4 T cells, suggesting a transcriptional restriction in this pathway (5). Though these data were obtained in absence of stimulation, such differences may suggest that the mechanisms regulating Th17 cell responses during the neonatal period lie upstream of RORγt. During Th17 cell differentiation, STAT3 cooperates with other cell signals, including TGF-β signaling, resulting from activation of two serine/threonine kinase transmembrane receptors: type I and type II TGF-β receptors (16). TGF-β signaling is mediated through the Small Drosophila Mothers Against Decapentaplegic (SMAD) proteins. Notably, SMAD2 and SMAD3 cooperate oppositely to regulate expression of the *RORC* gene, but also expression of the *IL17A* gene through SMAD4 (17).

Here, we sought to better understand the transcriptional and molecular events regulating the lack of Th17 cell response in human neonatal CD4 T cells. To this end, we directly compare neonatal and adult naïve CD4 T cells in an unbiased genome-wide gene expression analysis. Our analyses uncover major developmental changes in gene networks between adult and neonatal Th17 cells, unexpected from cells of the same lineage. We show changes in basic cellular domains, but more notably, T cell-specific domains such as Th differentiation, IL-7-dependent homeostatic proliferation and TGF-β signaling. These observations were functionally validated, by experiments revealing that the requirement for TGF-β during the differentiation of human Th17 cells is age-dependent. These experiments imply developmental changes in the activity of STAT3 and SMADs, and T cell biasing towards an immunoregulatory Th22 phenotype. Our findings, in primary human newborn T cells, support developmentally regulated transcriptional networks programmed to limit systemic Th17 cell responses during the newborn period. We propose that these changes at the same time increase newborns’ susceptibility to MC.

## Materials and Methods

### Blood Sample Collection

Cord and peripheral blood samples were collected in sodium heparin anti-coagulated Vacutainers (Becton Dickinson, Canada) from healthy term (>38-41 weeks of gestation) neonates born by cesarean section without labor at the Children’s & Women’s Health Centre of British Columbia (C&W) and healthy adults (range 20 to 40 years old). Samples were processed within 2 hours of collection.

### T Cell Purification, Stimulation and Cytokine Production

T cells were isolated by Fluorescent Activated Cell Sorting (FACS) or magnetic bead separation as detailed in **Supplemental Methods** (see also **Supplemental Table S1** for list of samples). Isolated T cells were stimulated with a 1:1 ratio of anti-CD3/CD28 beads (Thermo Fisher Scientific, DE, USA), as specified in RPMI1640 medium supplemented with 10% fetal bovine serum and 1% *Penicillin-Streptomycin*. During stimulation, the following cytokine polarizing conditions were used: Th0 (no exogenous cytokines), Th1: IL-12 (10 ng ml^−1^) and Th17: IL-6 (10 ng ml^−1^), IL-23 (10 ng ml^−1^), IL-1β (10 ng ml^−1^) ± TGF-β (3 ng ml^−1^) (all purchased from Peprotech, NJ, USA). IL-10 inhibition was achieved in presence of an anti-IL-10 (5 µg ml^−1^) and anti-IL-10 receptor, alpha subunit (5 µg ml^−1^) blocking antibodies (both from R&D systems, Ontario, Canada). STAT3 inhibition was achieved using Stattic (10 µM, Cayman Chemical, MI, USA). For T cell stimulation in the presence of antigen-presenting cells, neonatal or adult T cells were stimulated with a 1:1 ratio of allogeneic CD3-depleted neonatal or adult carboxyfluorescein succinimidyl ester (CFSE, Thermo Fisher Scientific, cat# C34554)-labeled antigen-presenting cells in the presence of anti-CD3 (OKT3; 0.5 μg ml^-1^) with or without an anti-CD28 antibody (1 μg ml^−1^; Invitrogen, # 11131D) for 6 days. For measures of T cell STAT3 activity, mononuclear cells were rested overnight at 37°C after thawing in RPMI1640 medium without serum, and stimulated with or without recombinant IL-6 (Thermo Fisher Scientific, USA) at a final concentration of 100 ng ml^-1^ for 15 minutes at 37°C. Procedures for intracellular and secreted cytokine and STAT3 experiments are detailed in **Supplemental Methods**.

### Gene Expression Analysis

The FACS isolation and data analysis strategies for gene expression experiments using naïve CD4 T cells data are depicted in **Supplemental Figure S1** (see **Supplemental Figure S2** for an example of the gating strategy that was used in FACS). RNA extraction, gene expression profiling, processing of whole-genome expression array data and Gene Set Enrichment analysis (GSEA) are detailed in **Supplemental Methods**. Thirteen genes (excluding STAT3) were chosen for confirmation by qPCR from separate neonatal and adult subjects (**Supplemental Figure S3**).

### STAT3 Transfections

A plasmid containing the human STAT3 and green fluorescent protein (GFP) cDNAs (**Supplemental Methods**) was transfected into magnetic bead-purified neonatal naïve CD4 T cells using a Lonza Nucleofector 2b device (Basel, Switzerland). Prior to transfections, the purity of these naïve T cells (>99.5% CD45RA^+^ cells) was verified by flow cytometry staining for the CD3, CD4 and CD45RA surface markers. After overnight cell culture at 37°C, dead cells were removed using a Dead Cell Removal kit (Miltenyi Biotech, # 130-090-101). The efficiency of transfection was assessed by green fluorescence protein expression measured by flow cytometry comparing STAT3-transfected and empty-vector-transfected cells. Transfected cells were stimulated and polarized as described above for 3 days. Cytokine expression data was normalized to the percentage of GFP-expressing cells in each sample.

### Statistics

Cytokine levels were compared using 2-tailed Mann-Whitney U (for independent groups) Wilcoxon test (for comparison of non-parametric data between stimulating conditions within same age groups) or paired t-tests (for comparison of parametric data between stimulating conditions within same age groups), or in some instances, 95% confidence intervals (for non-inferential, exploratory group comparisons where data was deemed to be normally distributed). Effects of age, stimulating cytokine conditions, and T cell or antigen-presenting cell age were assessed using 2-way ANOVAs as specified. Inferential group comparisons were adjusted for multiple testing using the Benjamini-Hochberg method and a 5% false-discovery rate, whenever indicated and as specified. P value of <0.05 was considered significant. All statistical analyses were conducted using GraphPad Prism 8 or 9 (San Diego, CA).

## Results

### Neonatal T Cells Are Inherently Biased Towards High IL-22 Cytokine Production

**Figure 1:**
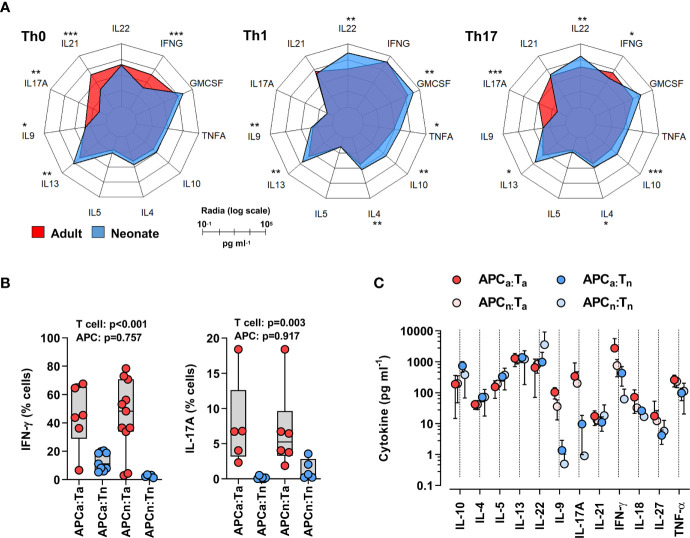
Neonatal T cells are inherently biased against Th17 responses independent of antigen-presenting cell signals. **(A)** Radar plots of cytokines produced by adult or neonatal naïve CD4 T cells 6 days post-stimulation (anti-CD3/CD28 beads) in Th0- (no exogenous cytokines), Th1- (IL-12) or Th17- (IL-1β, IL-6 and IL-23) polarizing conditions. Data are presented on a log-10 scale (pg ml^−1^) averaged from two separate sets of experiments that include a total of 6-12 subjects per cytokine polarizing condition per age group; P values for each cytokine were calculated by two-tailed Mann-Whitney U test using non-logarithmic values and correction was applied for multiple comparisons using the Benjamini-Hochberg method; *p < 0.05; **p < 0.01; ***p < 0.001; **(B)** Intracellular IL-17 and IFN-γ detection (by flow cytometry) in adult (red) or neonatal (blue) naïve CD4 T cells stimulated using soluble anti-CD3 antibody (OKT3) for 6 days in presence of carboxyfluorescein succinimidyl ester (CFSE)-labeled allogeneic neonatal (N) or adult **(A)** antigen-presenting cells (APC_a_ = adult APCs, APC_n_ = neonatal APCs, T_a_ = adult T cells, T_n_ = neonatal T cells). Data are aggregated from 11 independent experiments each using 2 freshly collected (same day) neonatal and adult samples, with p values for the effect of T cell or APC age on cytokine response, using a 2-way ANOVA; **(C)** Corresponding cytokine measurement in supernatants (ELISA); means with 95% CIs.

To understand age-specific T cell differentiation biases, cord blood (neonatal) and healthy adult peripheral blood naïve CD4 T cells (CD3^+^CD4^+^CD45RO^-^CCR7^+^CD25^-^) were isolated by Fluorescent Activated Cell Sorting (FACS) and stimulated using anti-CD3/CD28 beads for 6 days in the presence of either Th17-polarizing (IL-1β, IL-6 and IL-23), Th1-polarizing (IL-12), or no cytokines (Th0). As shown in **Figure 1A**, neonatal T cells stimulated in Th0 conditions produced low levels of Th1 (interferon-γ, IFN-γ) and Th17 (IL-17 and IL-21) cytokines compared to adult T cells. Notably, an abundance of IL-10, IL-13, Granulocyte-Macrophage Colony Stimulating Factor (GM-CSF), and also IL-22, was observed in Th1- or Th17-stimulated neonatal T cells (**Figure 1A**). These results confirm a Th2 bias, but further reveal another inherent neonatal T cell bias towards IL-22 rather than IL-17 production. Flow cytometry experiments by intracellular cytokine staining showed that IL-22-producing cells were distinct from IL-10-producing cells, and largely non-IL-17-producing (**Supplemental Figure S4**).

Neonatal antigen-presenting cells, particularly dendritic cells, make copious amounts of IL-1β, IL-6, and IL-23 (18). To determine to what extent the poor Th17 cell differentiation of neonatal T cells might be reversed by signals from neonatal antigen-presenting cells, we stimulated neonatal or adult T cells (using anti-CD3) in the presence of allogeneic adult or neonatal (cord blood) CD3-depleted mononuclear cells (without exogenous cytokines). Of note, this crossover experimental design allowed the mitigation of effects due to expected age-related differences in the proportion of *bona fide* antigen-presenting cells among blood mononuclear cells (19, 20). In these conditions, neonatal T cells still produced substantially less IL-17 and IFN-γ than adult T cells, in addition to reduced levels of IL-9, regardless of whether neonatal or adult antigen-presenting cells were used (**Figures 1B, C**; **Supplemental Figure S5**). Notably, neonatal T cells showed greater IL-22 production in the presence of neonatal antigen-presenting cells (**Figure 1C**). Neonatal antigen-presenting cells generally express lower levels of the CD28 ligands B7.1/2, and therefore may generally provide poor CD28 co-stimulation (8). Co-stimulation through ICOS enhances IL-22 production (21). To determine whether the lack of antigen-presenting cell CD28 co-stimulation overall could play a role here, experiments were repeated in the presence or absence of an anti-CD28 antibody. Differentiation of neonatal T cells in the presence of an anti-CD28 antibody further decreased Th17 cell responses and alternatively promoted increased IFN-γ production in neonatal T cells, regardless of the antigen-presenting cell used (**Supplemental Figure S6**).

### Unbiased Transcriptome Analyses Reveal a Th22 Bias and Altered TGF-β Signaling in Neonatal T Cells

**Figure 2:**
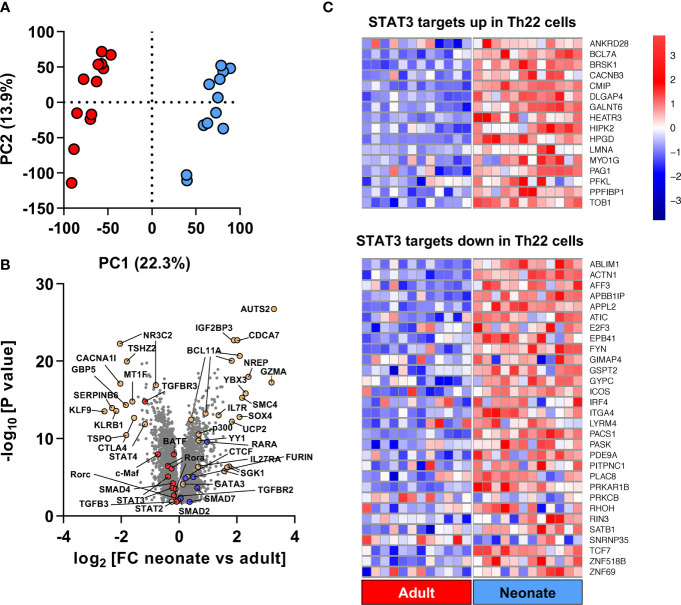
Genome-wide gene expression comparison identifies a unique developmental Th22 signature at baseline in naïve neonatal CD4 T cells. **(A)** Principal Component Analysis using above-intensity gene expression probes between unstimulated adult (red) and neonatal (blue) naïve CD4 T cells; **(B)** Volcano plot of fold-change and unadjusted p values of genes expressed differentially by neonatal and adult naïve CD4 T cells. TGF-β- and Th17-related genes with higher expression in adults are shown in red whereas genes with higher expression in neonates are shown in blue; **(C)** Heatmap of genes overlapping between STAT3 leading edge genes from the Chromatin Immunoprecipitation Enrichment Analysis (ChEA) database (**Supplementary Table S5**), and upregulated/down-regulated genes in Th22 cells compared to Th1, Th2 and Th17 cells based on (22). Red and blue colors in heat map represent Z-score for upregulated and downregulated genes in neonates compared to adults, respectively.

To investigate the transcriptional basis underlying the poor Th17 cell differentiation of human neonatal T cells, we undertook a genome-wide gene expression analysis comparing FACS-isolated naïve CD4 T cells (CD3^+^CD4^+^CD45RO^-^CCR7^+^CD25^-^) *ex vivo* from 12 neonatal (cord blood) and 12 healthy adult subjects. Gene expression profiles showed strong separation between the two age groups (**Figure 2A**), exposing a large number of differentially expressed genes (n=5,976; FDR <5%), corresponding to ~46% of the 12,909 detectably expressed unique annotated transcripts (**Supplemental Table S2**). One third (n=2,282) of the differentially expressed genes showed greater than 1.2-fold expression change between neonatal and adult T cells. Gene Ontology analysis mapped differentially expressed genes to broad cellular domains regulating cell cycle, post-transcriptional events and purine nucleoside monophosphate metabolism (**Supplemental Figure S7**, **Supplemental Table S3**).

Overall, the gene expression profile of neonatal T cells was, expectedly, consistent with Th2 pre-commitment, as evidenced by increased baseline expression of *GATA3* and decreased expression of Th1-regulating genes such as *IL12RB1*, *IFNGR2 and STAT4* (**Figure 2B**; **Supplemental Figure S8**). Expression of other Th2-regulating genes, such as SGK1 (a positive regulator of Th2 cells) (23) and SATB1 (a negative Th17 regulator) (24) increased in neonatal T cells. The configuration of gene networks also supported a neonatal T cell bias against Th17 cell differentiation, as shown by a reduced expression of *BATF* and *RORC*, and the *SGK1* genes that drive the ratio of Th17 and Treg cells towards pathogenic Th17 responses (25, 26). Gene Set Enrichment Analysis (GSEA) identified a Th22 gene signature within STAT3 targets in neonatal T cell, supporting Th22-biased neonatal T cell responses (**Figure 2C**).

**Figure 3:**
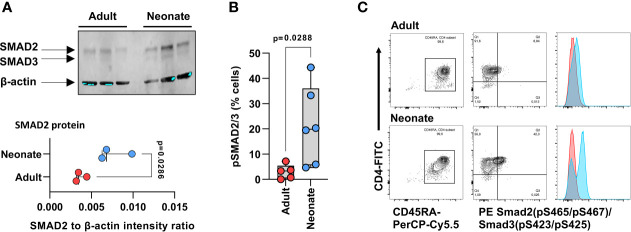
Expression and activity of SMAD2 and SMAD3 in naive neonatal and adult CD4 T cells. **(A)** Western blot detection of native SMAD2 and SMAD3 in magnetic bead-purified neonatal and adult naïve CD4 T cells (3 independent samples per age groups), and corresponding signal intensity for SMAD2 quantification relative to β-actin (lower panel). p value by two-tailed unpaired t-test; **(B)** SMAD2 and SMAD3 phosphorylation at serine residues 465 & 467, and serine residues 423 & 425 sites, respectively, by flow cytometry, in isolated naïve CD4 T cells (gated on CD4^+^CD45RA^+^ cells) after one hour incubation with TGF-β. p value also by two-tailed unpaired t-test; **(C)** Representative flow cytometry data in **(B)**, with histograms for cells incubated with TGF-β in blue and histograms in absence of TGF-β in red.

GSEA also revealed enrichment in other major gene sets regulating T cell biology: namely, Th differentiation and IL-7-dependent homeostatic proliferation, and most notably, TGF-β signaling (**Supplemental Figure S8**; **Supplemental Table S4**). TGF-β signaling-regulating genes such as the TGF-β receptors *TGFBR3*, *TGFBR2*, Furin, and also the *Small Drosophila Mothers Against Decapentaplegic* 2 (*SMAD2*), *SMAD4* and *SMAD7* were among the key differentially expressed genes. Expression of the *SMAD4* gene was decreased, whereas expression of the TGF-β-signaling inhibitor *SMAD7* (16) was increased, in neonatal T cells (**Figure 2B**; **Supplemental Table S2**). Even though we were unable to detect a differential expression of the *SMAD3* gene, the protein expression and activity of both SMAD2 and SMAD3 were increased in neonatal T cells (**Figure 3**). Additionally, GSEA showed enrichment in the expression of target genes for both SMAD2 and SMAD3 (**Supplemental Figure S9**; **Supplemental Table S5**). Altogether, these data support major developmental changes in key regulators of TGF-β signaling between neonatal and adult naive CD4 T cells.

### The Requirement for TGF-β During the Differentiation of Human Th17 Cells Is Age-Dependent

**Figure 4:**
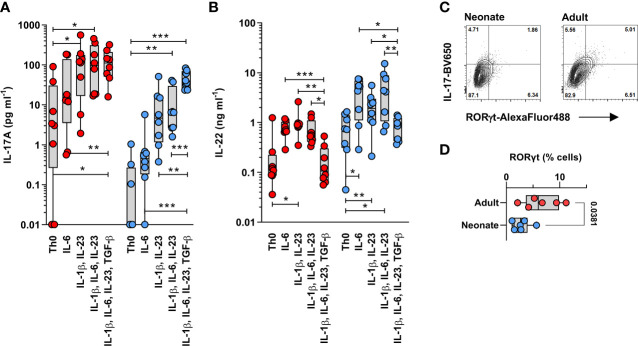
Neonatal T cells are skewed towards high IL-22 production and differ from adult T cells in their TGF-β during Th17 cell differentiation. IL-17 **(A)** and IL-22 **(B)** were measured in adult (red) and neonatal (blue) magnetic bead-isolated naïve CD4 T cells stimulated using anti-CD3/CD28 beads for 6 days in the presence of the indicated cytokine combinations. Data are combined from 8 separate experiments using cells from different subjects per age group, with cytokines measured in a single batch/assay; undetectable cytokines are shown as 0.01 pg ml^-1^; IL-17 **(A)** and IL-22 **(B)** production were significantly different between neonatal and adult T cells, as assessed using a 2-way ANOVA (age effects: p =0.023 for IL-17 and p =0.002 for IL-22); where statistical significance for the differences in cytokine stimulation conditions within age groups are shown after adjusting for multiple comparisons using a 5% false-discovery rate; *p < 0.05; **p < 0.01; ***p < 0.001; **(C)** Representative gating (on live cells) of RORγt expression in IL-17-producing cells from neonatal and adult naïve CD4 T cells differentiated for 6 days in the presence of IL-1β, IL-6, IL-23 and TGF-β, followed by a 5-hour PMA/Ionomycin stimulation (to bring up IL-17 expression), and **(D)** cumulative data from 6 independent neonatal and adult T cell samples.

The changes in TGF-β signaling between adult and neonatal T cells, at the transcriptome level, prompted further analyses to determine whether this could result in an age-dependent requirements for TGF-β signaling during human Th17 cell differentiation. To this end, magnetic bead-purified adult and neonatal naïve CD4 T cells were stimulated with anti-CD3/CD28 beads in the presence of combinations of IL-1β, IL-6, IL-23 and TGF-β cytokines, measuring IL-17A (**Figure 4A**) and IL-22 (**Figure 4B**) after 6 days. This experiment yielded three main observations: first, neonatal T cells produced much less IL-17, but substantially more IL-22 compared to adult T cells regardless of the cytokine stimulating conditions. This bias was confirmed by showing reduced RORγt expression in neonatal T cells (**Figures 4C, D**). Second, cytokine requirements somewhat differed between neonatal and adult naïve T cells during Th17 cell differentiation: IL-1β and IL-23 appeared sufficient in adult T cells, whereas IL-6 and TGF-β were additionally required to induce maximal IL-17 production in neonatal T cells. Exogenous TGF-β also enhanced IL-17 production in neonatal, but not in adult T cells. Third, adult T cells produced more IL-22 in the presence of IL-1β and IL-23, but IL-6 was essential for maximal IL-22 production by neonatal T cells; however, TGF-β suppressed this IL-22 production in both age groups (**Figures 4A, B**). This comparative experiment clearly identified age-related differences in the requirement for TGF-β during Th17 cell differentiation.

### Reduced *STAT3* Limits the Th17 Cell Differentiation of Neonatal T Cells

**Figure 5:**
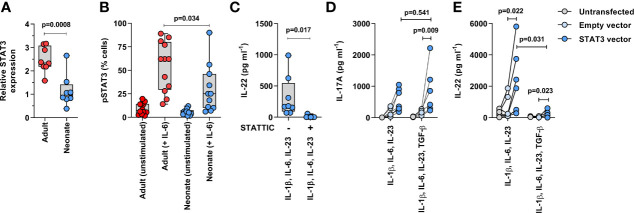
Reduced *STAT3* expression limits neonatal Th17 cell differentiation. **(A)** qPCR confirmation of *STAT3* gene expression in 8 neonatal and 8 adults naïve CD4 T cell samples (normalized on *GAPDH;* p value by unpaired T test); **(B)** STAT3 phosphorylation (intracellular flow cytometry) measured in neonatal and adult naïve CD4 T cells (gated on CD3^+^CD4^+^CD45RA^+^ cells) after 15 minutes stimulation with IL-6 (100 ng ml^−1^). Data are combined from 6 donors per age group in 2 separate experiments (p value by unpaired T test); **(C)** IL-22 production in neonatal T cells after 3 days of stimulation (anti-CD3/CD28 beads) in the presence of IL-1β, IL-6 and IL-23, with or without STAT3 inhibition (Stattic, 10μM); **(D)** IL-17 and **(E)** IL-22 production (normalized to %GFP+ live cells) in non-transfected, control (empty) GFP vector- and STAT3/GFP vector-transfected neonatal naïve CD4 T cells stimulated for 3 days (anti-CD3/CD28 beads) with IL-1β, IL-6 and IL-23 ± TGF-β; data are from 7 independent experiments, with lines connecting cells from the same blood donations; p value by paired t-test or Wilcoxon test.

Lastly, we aimed to determine whether STAT3 activity was reduced in neonatal T cells and whether this could limit their Th17 cell differentiation capacity. First, reduced *STAT3* gene expression in neonatal T cells was confirmed by qPCR in another set of neonatal and adult naïve CD4 T cell samples (**Figure 5A**). We also confirmed that STAT3 activity was reduced in neonatal T cells, as evidenced by reduced STAT3 phosphorylation at tyrosine residue 705 (Y705) in the presence of IL-6 (**Figure 5B**, **Supplemental Figure S10**), despite adult-like expression levels of the IL-6 receptor (**Supplemental Figure S11**). However, production of IL-22 by human T cells being also largely STAT3-dependent (27), additional experiments confirmed that neonatal T cells show some functionally relevant residual STAT3 activity, as indicated by a decrease in IL-22 production in the presence of a STAT3 inhibitor (**Figure 5C**). Importantly, transient overexpression of STAT3 (**Supplemental Figure S12**) increased neonatal IL-17 production when cells were stimulated with anti-CD3/CD28 in the presence of IL-1β, IL-6, and IL-23 (**Figure 5D**). These data support an important role for altered STAT3 signaling in limiting neonatal Th17 cell differentiation. Notably, STAT3 overexpression also increased IL-22 production, which at the same time remained suppressible by TGF-β (**Figure 5E**). Finally, since phosphorylation of STAT3 can occur through the IL-10 receptor, and IL-10 inhibits Th17 cell differentiation and is abundantly expressed by neonatal T cells (**Figure 1**) (28), we wanted to exclude an inhibitory effect of this cytokine on IL-17 production. However, blocking of IL-10 and its high affinity receptor IL-10R1 did not detectably increase IL-17 production in neonatal T cells (**Supplemental Figure S13**). Thus, these experiments identified STAT3 as an important developmental modulator of Th17 cell differentiation in human neonatal T cells.

## Discussion

This study revealed surprisingly large differences in gene networks between neonatal and adult naïve CD4 T cells *ex vivo*, suggesting that these two cells represent distinct lineages (29). These results further show that the biology of human naïve T cells is fundamentally different at these two life stages, implying that the human adaptive immune system is programmed to complete its maturation during post-natal life when it is most exposed to foreign antigens. Neonatal CD4 T cells exhibited non-stereotypic Th17 cell responses characterized by a bias towards high production of the immunoregulatory IL-10 and IL-22 cytokines. Unbiased transcriptome and functional experiments implicate major developmental changes in gene networks regulating STAT3 and TGF-β signaling. To the best of our knowledge, this is the first study comparing the Th17 cell differentiation cytokine requirements between neonatal and adult CD4 T cells directly side-by-side. Results showed that the requirement for TGF-β during human Th17 cell differentiation is age-dependent, and manifested mainly with cord blood, but not adult peripheral blood. This important nuance mandates a re-interpretation of previous STAT3/TGF-β functional and gene network data, requiring that the age of the T cell donor be more carefully considered in the interpretation of these studies (15, 30).

Pathological increase in Th17 cell response during the newborn period has been linked to necrotizing enterocolitis - a serious gastrointestinal disease manifesting exclusively in newborns (31). In a neonatal mouse model, systemic blockade of IL-17 reduced mortality from polymicrobial sepsis, suggesting that a high IL-17 production is also disadvantageous during neonatal sepsis (32). During this period of life, naïve CD4 T cells may preferentially adopt an immunoregulatory Th22 phenotype, rather than a stereotypic Th17 phenotype, in order to help maintain local immune homeostasis (33). IL-17 and IL-22 are two cytokines that can be produced by *bona fide* Th17 cells. Similar to IL-17, IL-22 also acts on epithelial mucosal cells in the skin, liver, gut, and lung (34). IL-22 is a member of the IL-10 family of anti-inflammatory cytokines that target epithelial cells. However, in contrast to IL-17, IL-22 has protective effects in the gut, promoting intestinal epithelial regeneration, enhancing mucin production and the post-translational fucosylation of sugar moieties on mucin proteins, which promotes beneficial gut microbial colonization) (34, 35). Systemically, IL-22 also enhances antibody-mediated immunity and pathogenic bacterial clearance *via* the iron scavenger hemopexin (36, 37). In the light of these findings we postulate that the preferential bias of circulating neonatal T cells towards IL-22 is essential to limit the systemic consequences of mucosal inflammation during *de novo* gut colonization with commensal microbes.

Given the phenotype of humans with genetically impaired Th17 cell responses, it is tempting to establish a link between the developmental lack of neonatal Th17 cell differentiation and the increased susceptibility of neonates to MC. On the other hand, humans with mutations in the IL-17 pathway also exhibit increased susceptibility to *Staphylococcus aureus* folliculitis, which is not commonly seen in neonates. A possible explanation may lie in the ability of neonatal T cells to produce high levels of IL-10 regardless of their Th differentiation conditions (**Figure 1A**). Supporting this contention, we note that neonates who have a genetic defects in *IL10RB* also show an increased susceptibility to *Staphylococcus aureus* folliculitis (1, 38, 39). Thus, production of IL-10 by neonatal Th17 cells might be protective against this condition despite their developmental lack of IL-17 production. Thus, we also conclude that the phenotype of neonatal T cells resembles the immunoregulatory phenotype of IL-10-producing *tissue-resident Th17 cells* (40) more than the phenotype of T cells from humans with genetic IL-17 defects (38).

Data reveal age-related differences in STAT3 activity and in the requirements for the TGF-β cytokine during Th17 cell differentiation in humans. Gene expression data closely implicate differential activity in SMADs. Mechanistic studies are required to elucidate how these changes operate to alter TGF-β signaling in neonatal T cells. However, we can speculate on this matter here: on the one hand, SMAD2 phosphorylation enhances *RORC* gene expression (41). On the other hand, SMAD4 can indirectly suppress *RORC* expression by interacting with SKI, a transcriptional repressor that controls histone acetylation/de-acetylation at the *RORC* locus (16, 42). TGF-β signaling causes the degradation of SKI, to help SMAD4 suppress the *RORC* locus (16, 42). Therefore, our data support a model whereby increased SMAD2 activity in the context of reduced *SMAD4* renders naïve neonatal CD4 T cells more dependent on TGF-β signaling during the Th17 cell differentiation, despite lower STAT3 activity. In addition, mechanisms common to both neonatal and adult T cells, such as the transcription factor c-Maf that suppresses IL-22 production by binding directly to its proximal gene promoter region (43), may explain how TGF-β suppresses IL-22 production in both of these cell types.

Despite limited STAT3 activity in neonatal T cells, STAT3 expression levels appear sufficient to produce a robust IL-22 response regardless of the IL-17 response. IL-21 also provides positive feedback to amplify the precursor frequency of Th17 cells along with TGF-β (44–46). Together with expression of the transcriptional regulator of Th22 cells, *aryl hydrocarbon receptor* (*AhR*), IL-21 can also activate STAT3 to induce IL-22, but not IL-17, production (47). However, the comparable IL-21 expression between neonatal and adult T cells in Th1 or Th17-polarizing conditions does not support a developmental effect through this cytokine. Intracellular flow cytometry cytokine staining experiments showed that the majority of cells between day 5 and day 21 produce either IL-17 or IL-22 rather than both cytokines. However, the interpretation of these preliminary experiments was limited by the differences in timing of intracellular detection for these two cytokine. Further studies are needed to determine whether a small subset of cells can express both cytokines, under specific T cell receptor/cytokine stimulatory conditions.

Studies are also needed to understand how human T cell gene network are regulated developmentally. Considering that the action of transcription-factor networks is context-dependent during the lineage differentiation of CD4 T cells (48), it is plausible that broad developmental-specific epigenetic changes underlie the changes in gene expression observed in this study. The implication of epigenetic changes regulating the network of active transcription factors, as well as the amount of TGF-β signaling forms the basis of the continuum of “classical” to “alternative” Th17 cells. This concept reinforces the notion that lineage-regulating transcription factors influence the final effector phenotype of Th17 cells, potentially through developmentally altered changes in chromatin accessibility (49).

Our study has limitations. First, it only examined cells from adult and cord blood, missing the developmental continuum between these two life stages. Other studies have shown that Th17 cell differentiation remained low up to 3 months of age in infants, so we expect similar mechanisms to operate at least from birth until then (50). Another limitation is that the data were focused on conventional CD4 T cells, while Th17 responses in newborns may also be carried out by other cells in peripheral organs (e.g. the gut), such as γδ T cells or type 3 innate lymphoid cells (31, 51), or other T cell subsets in peripheral tissues (52). Finally, others have shown that peripheral blood neonatal T cells can produce vigorous Th17 responses when challenged with *Candida* extracts (53), so it would be important to study neonatal T cell responses in the context of MC in order to fully understand their susceptibility to this micro-organism. Nonetheless, studies like the current one provides important mechanistic insights into the regulation and function of conventional T cells during the neonatal period.

## Data Availability Statement

The datasets presented in this study can be found in online repositories. The names of the repository/repositories and accession number(s) can be found below: https://www.ncbi.nlm.nih.gov/geo/, GSE135467.

## Ethics Statement

The studies involving human participants were reviewed and approved by University of British Columbia Children’s & Women’s Research Ethics Board. Written informed consent to participate in this study was provided by the participants’ legal guardian/next of kin.

## Author Contributions

HR, ZS, AS, and PL designed the study. HR, ZS, AS, GB, KL, and RDS conducted the experiments. HR, ZS, AS, GB, and PL analyzed the data. PO and ZS designed and made the STAT3 construct. RPS provided reagents and resources. CR supervised the Illumina expression array experiments. HR, ZS, PO, and PL wrote the manuscript. All authors contributed to the article and approved the submitted version.

## Funding

HR was funded by a fellowship from the MITACS national research organization. ZS was supported by Graduate Studentship Award from BC Children’s Hospital Research Institute. AS was supported by a Child & Family Research Institute (CFRI) Graduate Studentship. GB was supported by a University of British Columbia Faculty of Medicine Award. RDS was supported by a BC Children’s Hospital Research Institute Summer Studentship. PL was supported by an Investigator Award from the BC Children’s Hospital Research Institute and a Career Investigator Award from the Michael Smith Foundation for Health Research (MSFHR). This research was funded by Canadian Institutes of Health Research grants (MOP-110938 and MOP-123478, to PML).

## Conflict of Interest

The authors declare that the research was conducted in the absence of any commercial or financial relationships that could be construed as a potential conflict of interest.
