# Supplementary material for: Neonatal T Helper 17 Responses Are Skewed Towards an Immunoregulatory Interleukin-22 Phenotype
Source: Front Immunol. 2021 May 3;12:655027. doi: 10.3389/fimmu.2021.655027 (PMC8126652; doi:10.3389/fimmu.2021.655027)
Supplement: Supplementary file 1 [file DataSheet_1.pdf]

SUPPLEMENTAL FIGURES

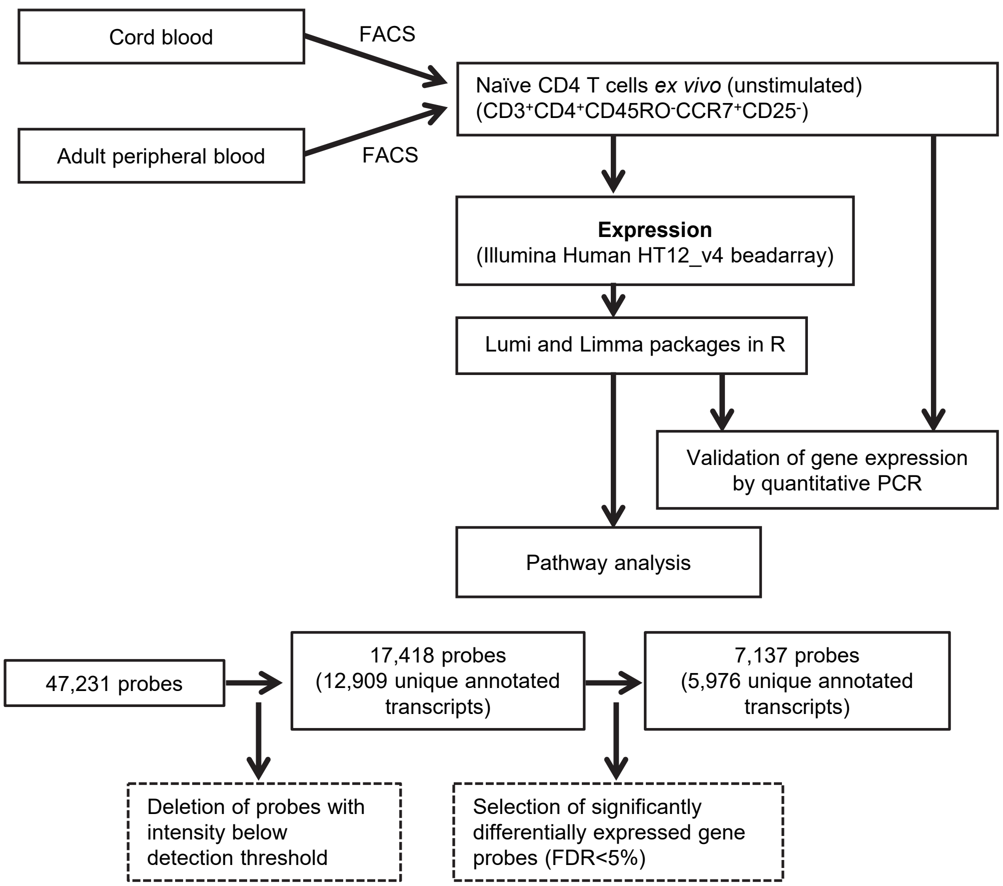

Supplemental Figure S1: Fluorescent Activated Cell Sorting (FACS)-isolation and analysis strategy for gene expression data comparing FACS-isolated neonatal and adult naive CD4 T cells; FDR: false-discovery rate.

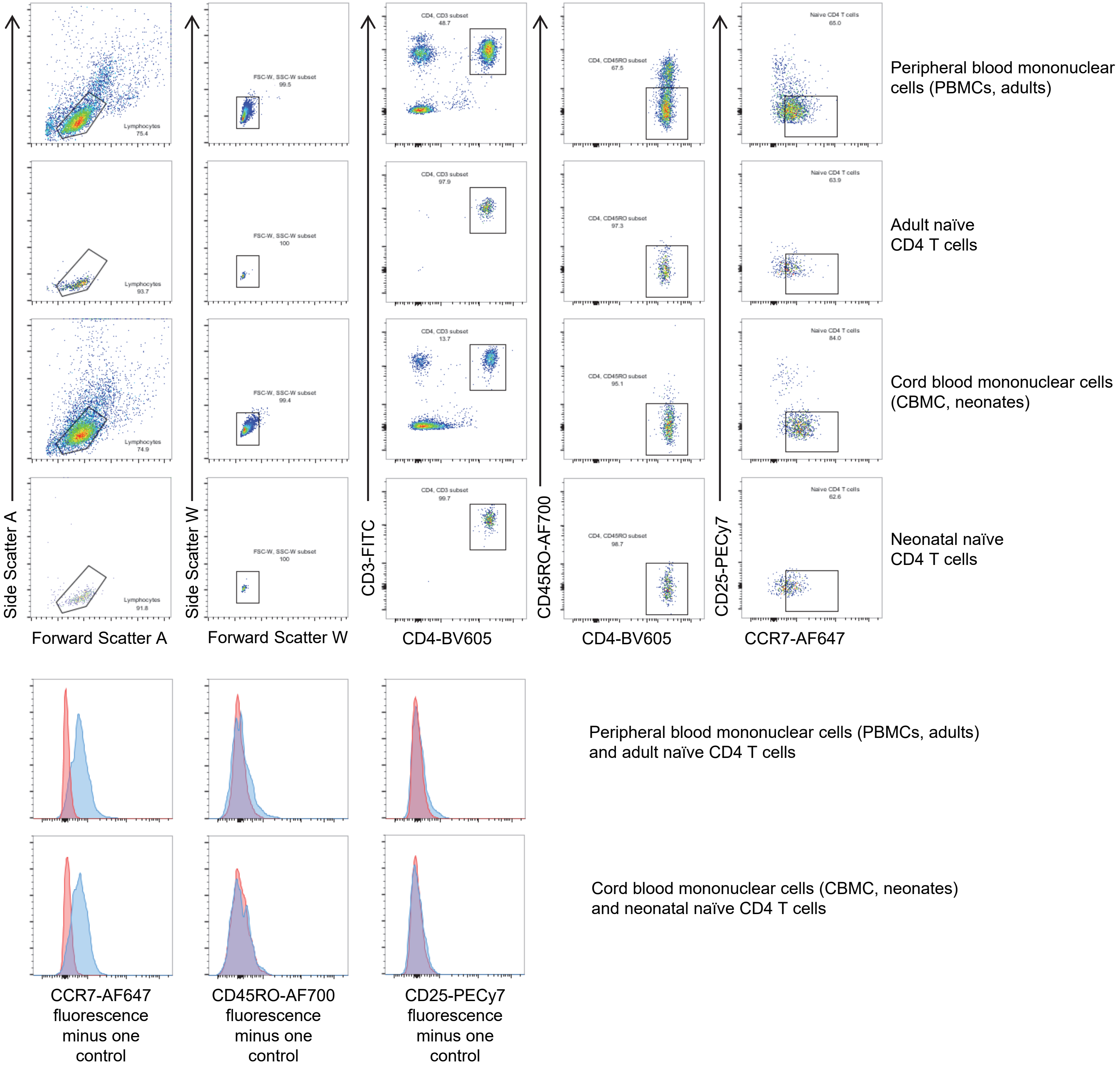

Supplemental Figure S2: Representative of gating strategy and flow cytometry analysis of naïve adult and neonatal CD4 T cells isolated via fluorescence activated cell sorting (FACS) method and used in Illumina genome-wide gene expression experiments. In the lower graphs, red overlays are Fluorescence minus one (FMO) controls.

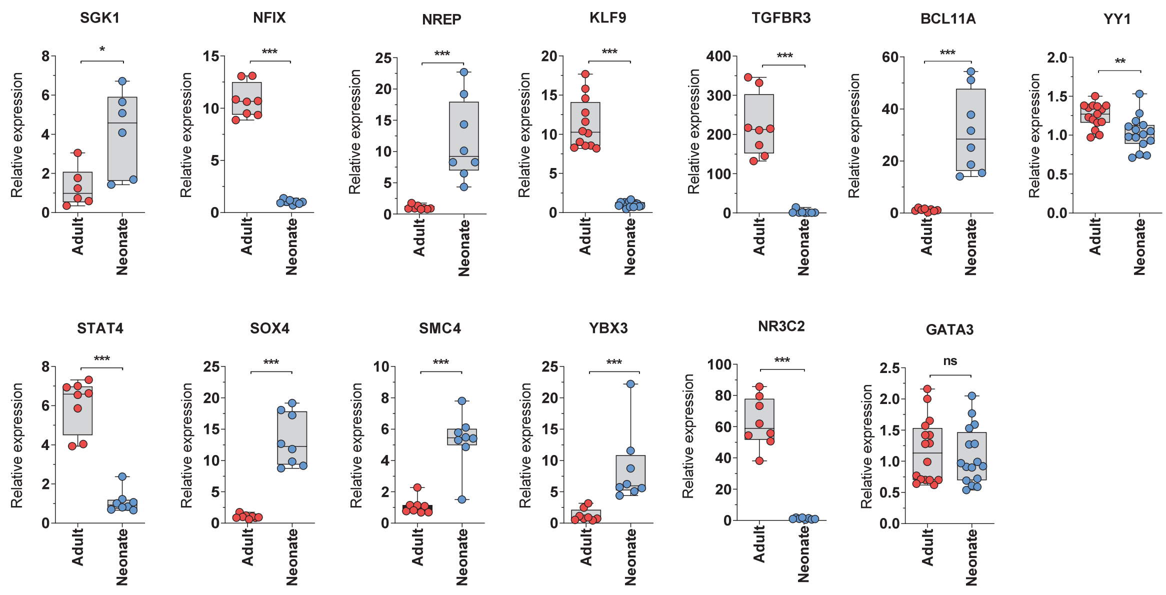

Supplemental Figure S3: PCR quantification of selected differentially-expressed genes between naïve neonatal and adult CD4 T cells. qPCR quantification of selected genes from 6 to 16 neonatal and adult FACS-isolated naïve CD4 T cell samples (bars represent median). *p<0.05; **p<0.01; ***p<0.001 by two-tailed Mann-Whitney U test.

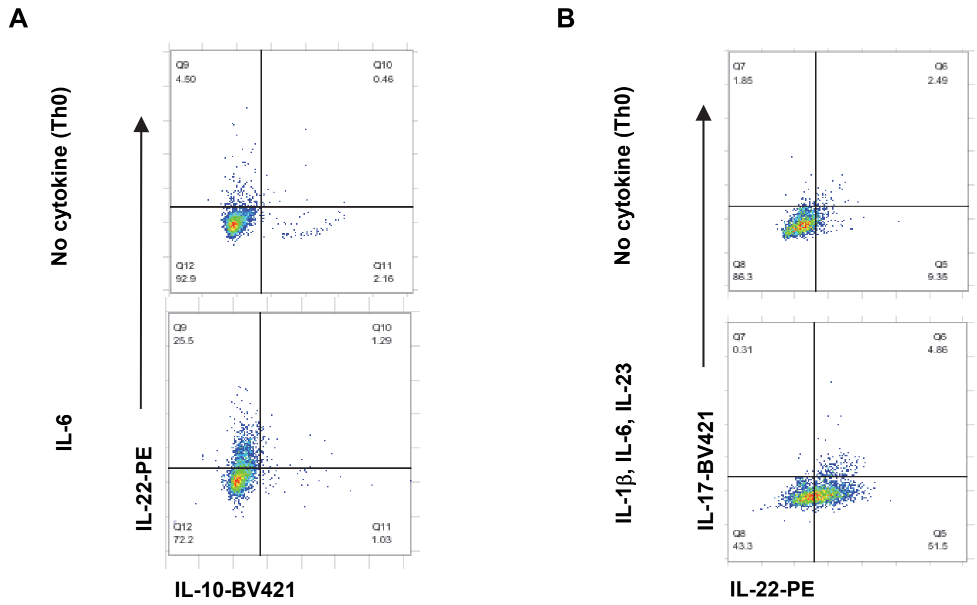

Supplemental Figure S4: Flow cytometry intracellular cytokine staining. Negatively-depleted, naïve neonatal CD4 T cells were stimulated for 6 days in the presence of no additional cytokine (Th0) or (**A**) IL-6 and stained for IL-22/IL-10, or (**B**) IL-1b, IL-6 and IL-23 and stained for IL-22/IL-17, after a 6-hour PMA/Ionomycin stimulation (gated on live, CD4-expressing cells).

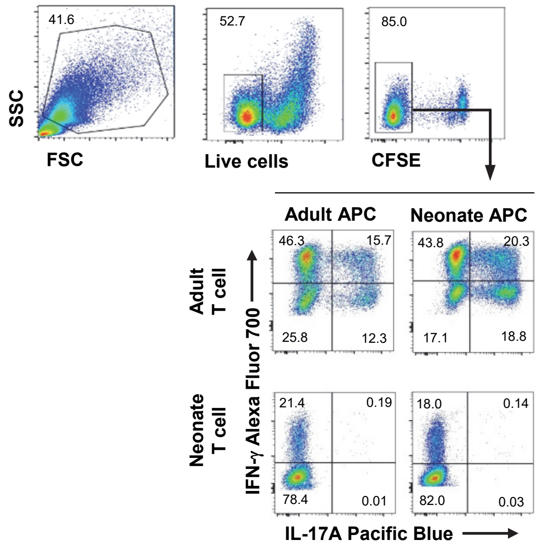

Supplemental Figure S5: Representative flow cytometry gating for intracellular IL-17 and IFN-γ expression in naïve adult or neonatal CD4 T cells stimulated with anti-CD3 (OKT3; 0.5 μg ml^{-1}) in the presence of allogeneic neonatal (N) or adult (A) CD3-depleted mononuclear cells (used as antigen presenting cells). Flow cytometry gate excludes carboxyfluorescein succinimidyl ester (CFSE)-labeled cells (antigen presenting cells).

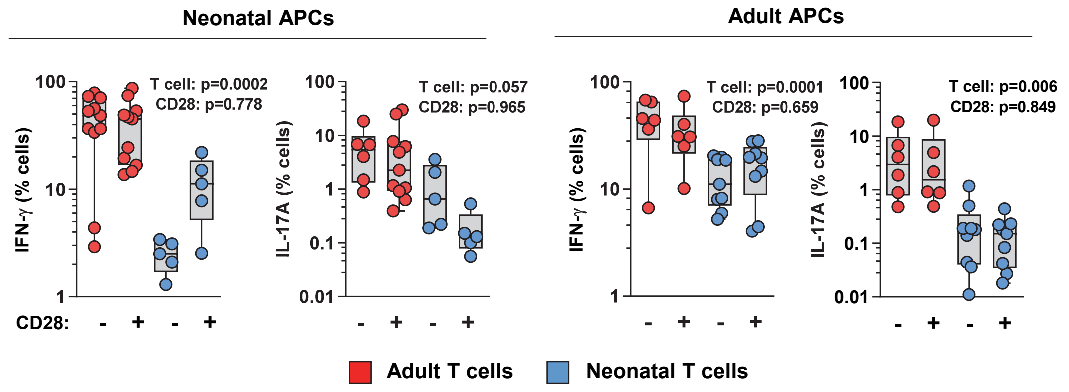

Supplemental Figure S6: Effect of CD28 co-stimulation on Th1/Th17 responses in adult or neonatal naïve CD4 T cells. Intracellular IL-17 and IFN-γ detection (by flow cytometry) in adult or neonatal naïve CD4 T cells stimulated using soluble anti-CD3 (OKT3) for 6 days in presence of carboxyfluorescein succinimidyl ester (CFSE)-labeled allogeneic neonatal (N) or adult (A) CD3-depleted mononuclear cells (used as APCs) with or without exogenous anti-CD28 antibody co-stimulation (1 μg ml^{-1}). P values represent effect from T cell and CD28 co-stimulation on cytokine responses, using a 2-way ANOVA.

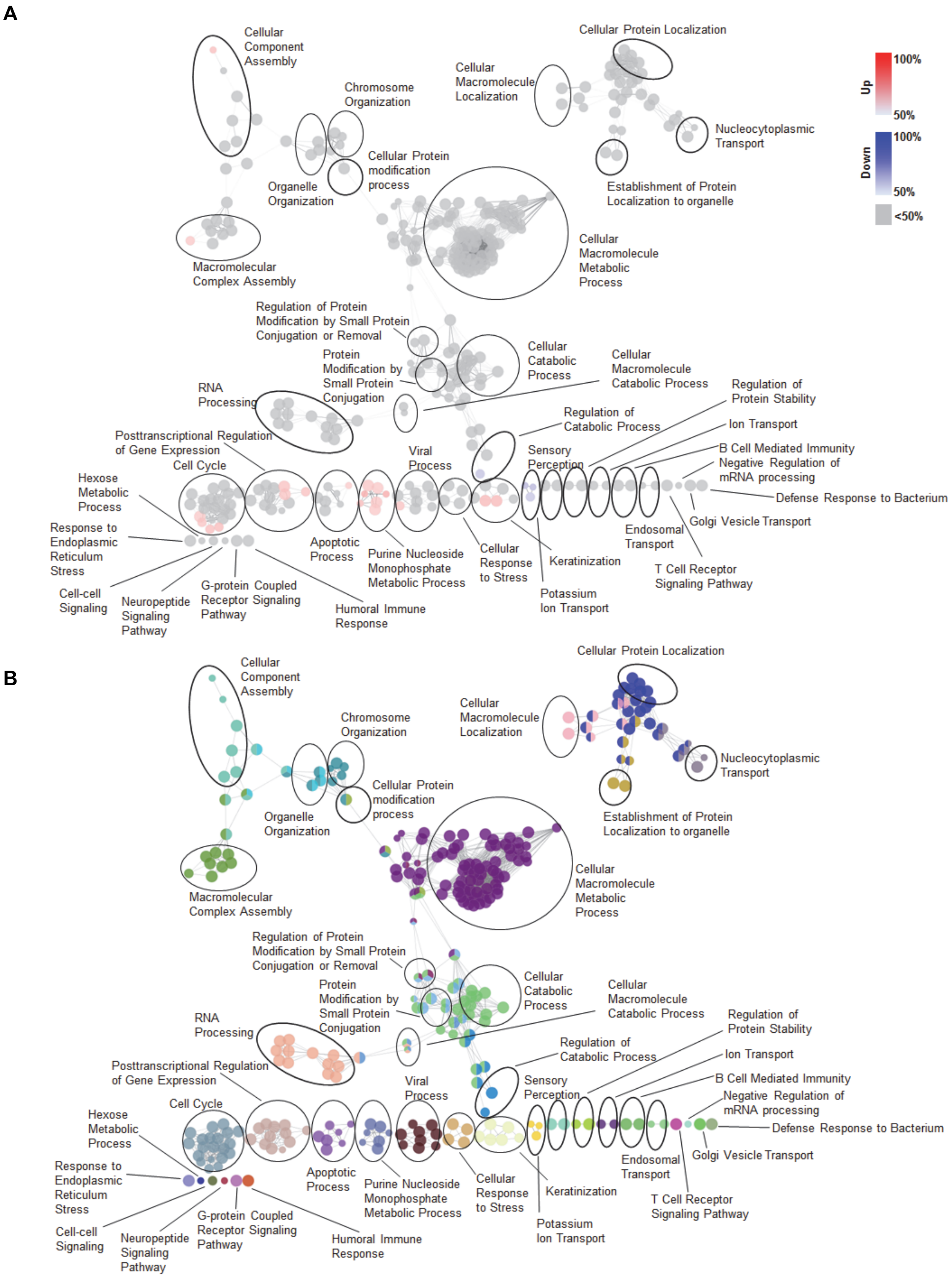

Supplemental Figure S7: Cytoscape representation of Gene Ontology pathways enriched with genes differentially expressed (FDR<5%) between neonatal and adult naïve CD4 T cells. In **(A)** red and blue colors represent groups with >50% enrichment between neonatal and adult T cells; Grey represents pathways that are less than 50% enriched. Cut-off p-value<0.05; In **(B)** each color represents a functional group (p-value<0.05).

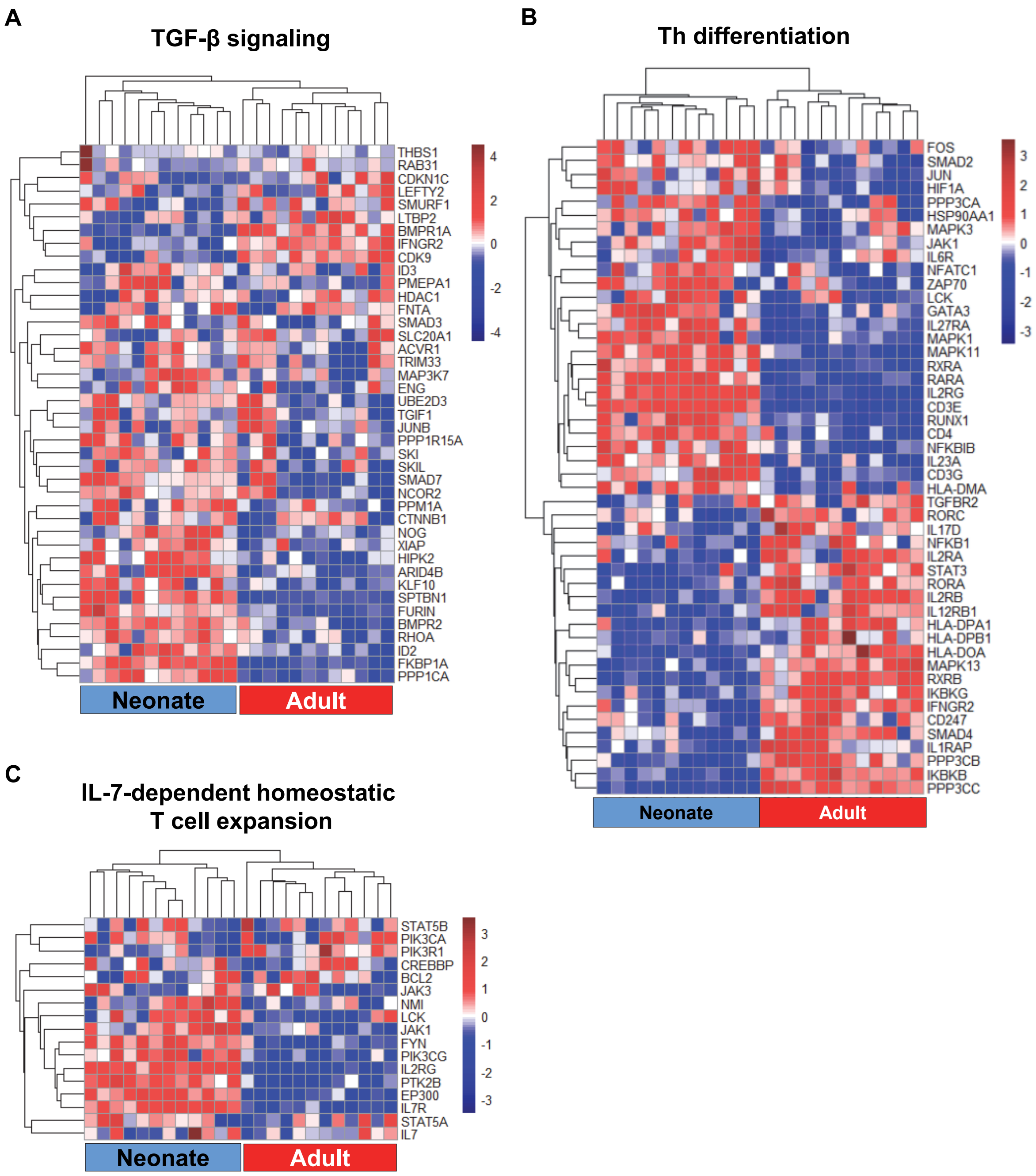

Supplemental Figure S8: Expression heatmap with hierarchical clustering of genes from Gene Set Enrichment Analysis between neonatal and adult naïve CD4 T cells. (**A**) TGF-β signaling (41 genes), (**B**) Th differentiation-related gene pathways (48 genes) and (**C**) IL-7-dependent homeostatic T cell expansion (17 genes). Red and blue colors show upregulated and downregulated genes in neonates compared to adults, respectively, on a Z-score scale.

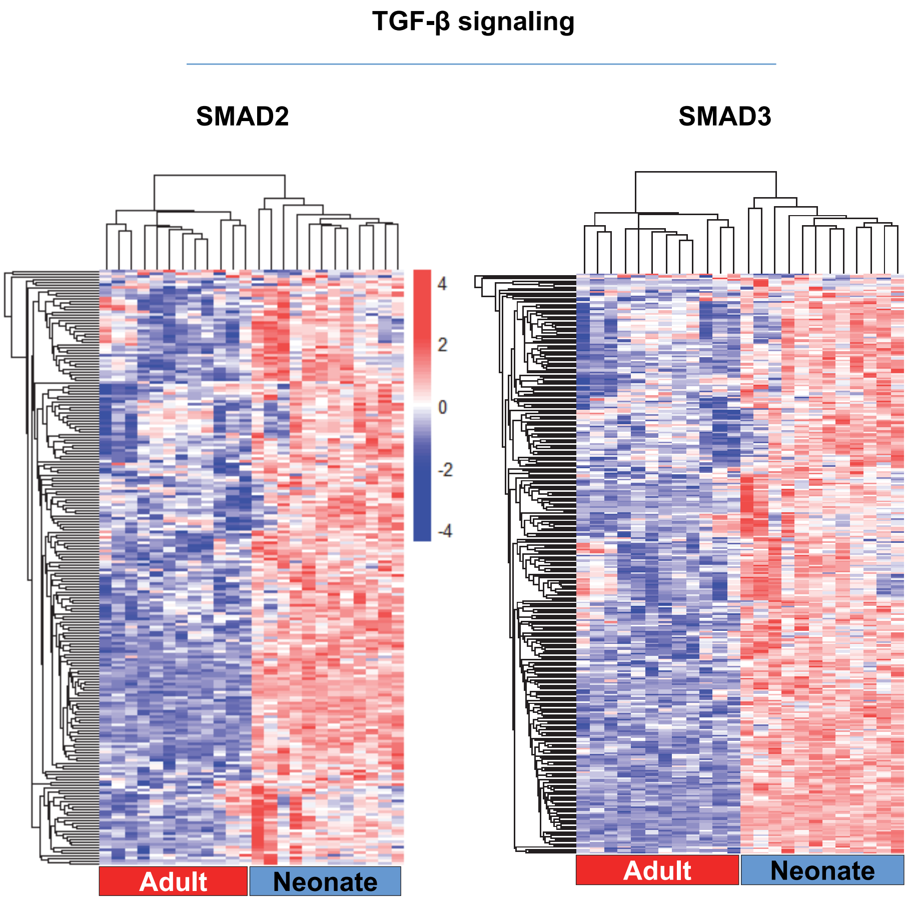

Supplemental Figure S9: Expression heatmap of leading edge genes for gene targets of SMAD2 and SMAD3. Leading edge genes of the gene set enrichment analysis of transcription factors in supplementary table S5 from the ChEA (Chromatin Immunoprecipitation Enrichment Analysis) database are presented. Red and blue colors show upregulated and downregulated genes in neonates compared to adults, respectively, on a Z-score scale.

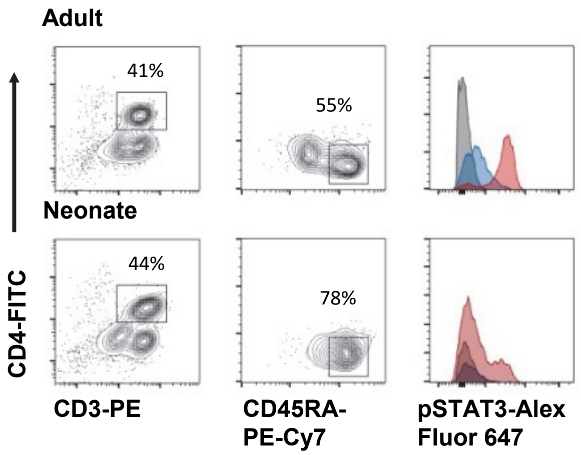

Supplemental Figure S10: Representative experiment and gating strategy for STAT3 phosphorylation (by flow cytometry) in neonatal and adult naïve CD4 T cells (gated on $CD3^+CD4^+CD45RA^+$ cells) after 15 minutes of stimulation with IL-6 (100 ng ml^{-1}). Colors represent fluorescence-minus one control (grey), unstimulated (blue) and stimulated (red) samples.

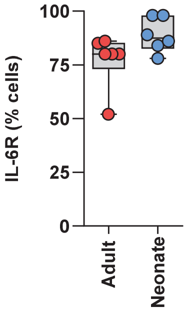

Supplemental Figure S11: IL-6 receptor is expressed on naïve neonatal CD4 T cells. Expression of IL-6 receptor, or CD126 was determined by flow cytometry on blood mononuclear cells (gated on $CD3^+CD45RA^+CCR7^+CD25^-$ cells).

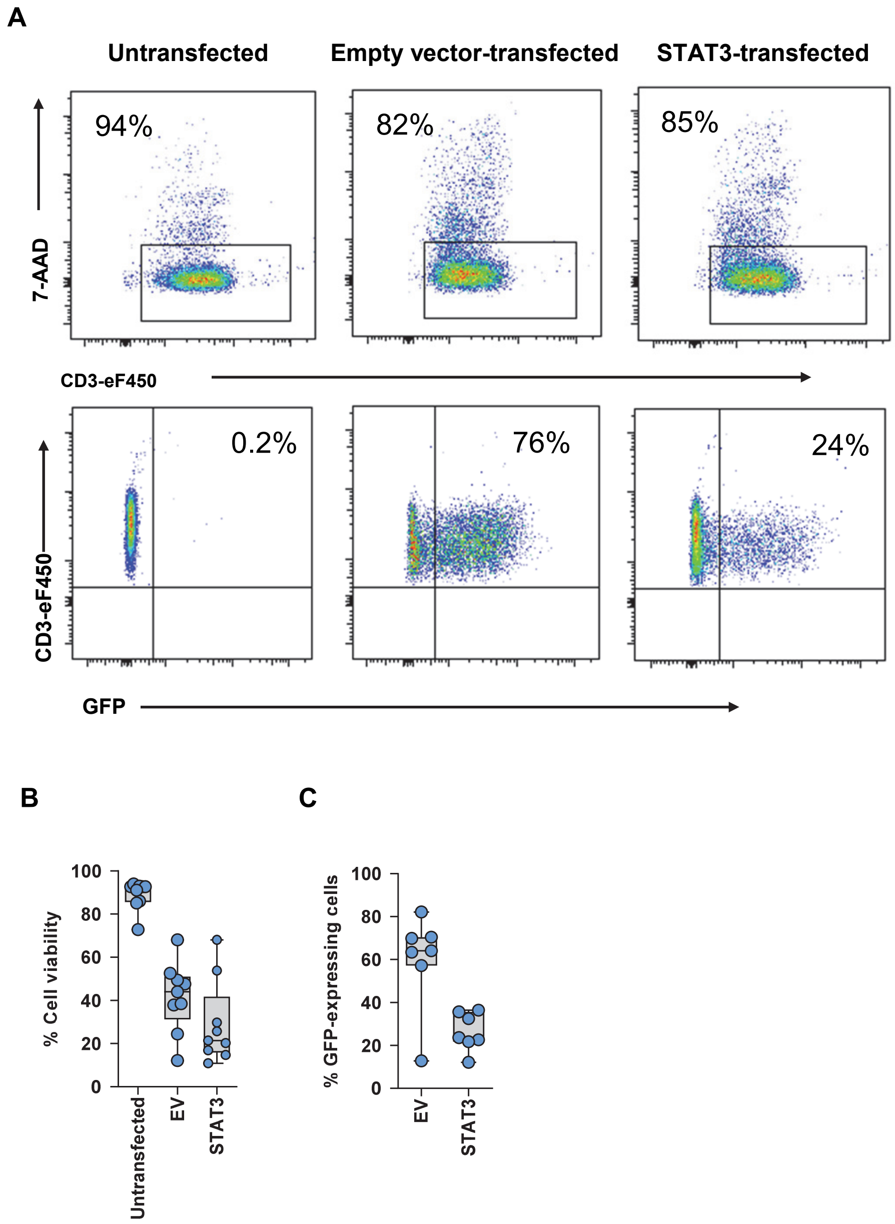

Supplemental Figure S12: Transfection of naïve neonatal CD4 T cells using STAT3-expressing plasmid vector (A) Representative cell viability (7-AAD) and transfection efficiency (GFP-positive cells) after transfection of neonatal naïve CD4 T cells with empty vector (EV) or STAT3-expressing vector; aggregated data for (**B**) cell viability and (**C**) GFP-expression from 9 separate transfection experiments using separate cord blood donors.

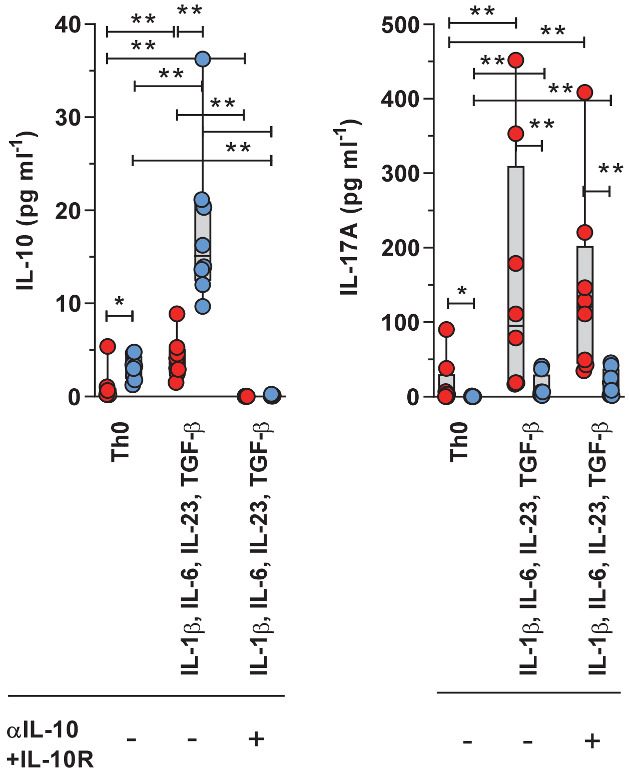

Supplemental Figure S13: Effect of IL-10 blocking on IL-17 production (ELISA) in neonatal and adult naïve CD4 T cells. T cells were stimulated with anti-CD3/CD28- coated beads for 6 days without exogenous cytokines (Th0) or in the presence IL-1β, IL-6, IL-23 and TGF-β ± anti-IL-10 and anti-IL-10 receptor antibodies. P values were calculated by paired Wilcoxon test (within age groups) or Mann Whitney (between age groups). Data are from one experiment with 8 subjects per group. *p<0.05; **p<0.01.

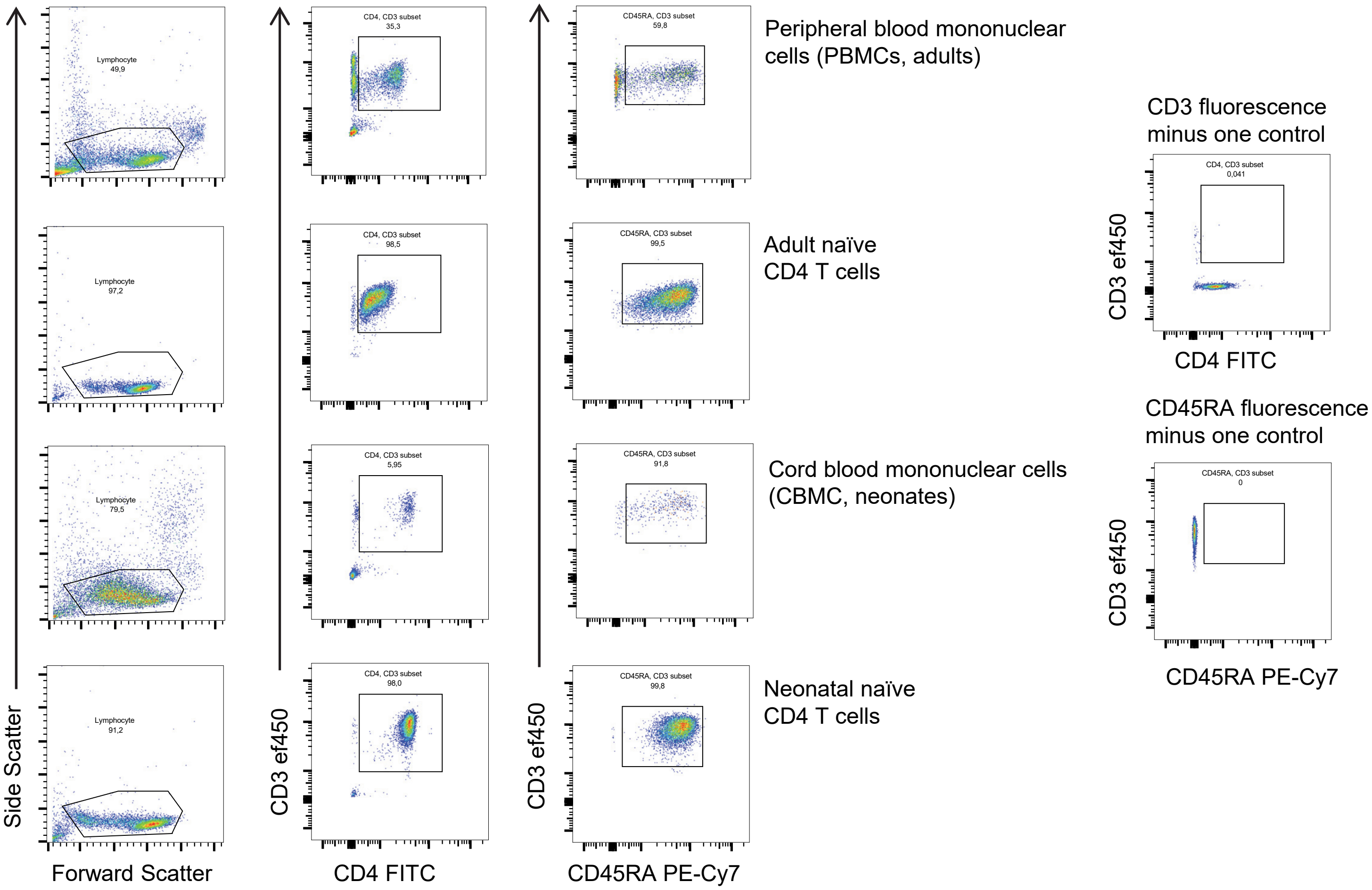

Supplemental Figure S14: Representative flow cytometry analysis of magnetic bead-purified naïve adult and neonatal CD4 T cells used to confirm ≥99.5% purity after staining for CD3, CD4, and CD45RA.
