# Supplementary material for: Neonatal T Helper 17 Responses Are Skewed Towards an Immunoregulatory Interleukin-22 Phenotype
Source: Front Immunol. 2021 May 3;12:655027. doi: 10.3389/fimmu.2021.655027 (PMC8126652; doi:10.3389/fimmu.2021.655027)
Supplement: Supplementary file 2 [file DataSheet_2.docx]

## Cells: Cord and peripheral blood mononuclear cells (BMCs) were extracted using Lymphoprep^TM^ (StemCell Technologies, Vancouver, Canada). Naïve T cells were isolated either by Fluorescent Activated Cell Sorting (FACS) on a BD FACSAria^TM^ II using anti-CD3 FITC (clone UCHT1; eBioscience, Canada) or PE (clone HIT3a; BD Biosciences, Canada), anti-CD4 BV605 (clone OKT4; BioLegend, CA USA), anti-CCR7 Alexa Fluor 647 (clone 3D12; BD Biosciences, Canada), anti-CD25 PE-Cy7 (clone M-A251; BD Biosciences, Canada), anti-CD45RO Alexa Fluor 700 (clone UCHL1; BioLegend, CA USA) with or without anti-CD235 FITC (clone 10F7MN; eBioscience, Canada) antibodies, or by negative depletion using EasySep™ Human Naïve CD4^+^ T Cell Isolation Kit magnetic beads (StemCell Technologies, Vancouver, Canada). The purity of bead-purified naïve CD4 T cells was further assessed by flow cytometry, in which cells were stained for CD3, CD4, and CD45RA to confirm ≥99.5% CD45RA^+^ T cells (Supplemental Figure S14). For gene expression analyses, FACS-isolated T cell pellets were immediately stored at -80 ºC after cell sorting. Antigen presenting cells were obtained by first depleting T cells from neonatal or adult BMCs, using the EasySep Human T cell isolation kit magnetic beads (StemCell Technologies, Vancouver, Canada); after confirming >99.5% depletion of CD3 cells, the remaining cells were then irradiated (5000 cGy) and labelled using carboxyfluorescein succinimidyl ester (CFSE) (Thermo Fisher, cat# C34554).

## Cytokine, IL-6 receptor, and STAT3 / SMAD activity: For intracellular cytokine measurements, T cells were re-stimulated for 6 hours with PMA (100 ng ml^−1^; Millipore/Sigma, Canada) and ionomycin (1 µg ml^−1^; Invitrogen, Canada) in the presence of Brefeldin A (10 µg ml^−1^), and then stained using the following fluorescence-conjugated antibodies: anti-CD4-BV605 (clone OKT4; BioLegend, USA), anti-IFN-γ-Alexa Fluor 700 (clone 4S.B3; BioLegend, USA), anti-CD14-V500 (for monocyte exclusion; clone M5E2; BD Biosciences, Canada), anti-IL-17A-BV421 (clone BL168, BioLegend, USA), anti-IL-10-BV421 (clone JES3-9D7, BioLegend, USA), anti-IL-17A-BV650 (clone N49-653, BD Biosciences, Canada) and/or anti-IL-22-PE (clone 2G12A41, BioLegend, USA). Secreted cytokines were measured from supernatants collected on day 6 post-stimulation, using a Th1/Th2/Th9/Th17 cytokine 18-plex assay (Thermo Fisher Scientific, cat#EPX180-12165-901); only T cell-relevant cytokines are shown.

## For IL-6 receptor measurements, BMCs were stained with the following fluorescence-conjugated antibodies: anti-CD3-eF450 (clone UCHT1; eBioscience, Canada), anti-CD45RA-PerCp-Cy5.5 (clone HI100; eBioscience, Canada), anti-CCR7-Alexa Fluor 647 (clone 3D12; BD Biosciences, Canada), anti-CD25-PE-Cy7 (clone M-A251; BD Biosciences, Canada), and anti-CD126-BB515 (clone M5; BD Biosciences, Canada).

## For STAT3 phosphorylation, stimulated mononuclear cells were fixed using pre-warmed Cytofix^TM^ buffer (BD Biosciences, Canada), permeabilized using Perm/wash buffer (BD Biosciences, Canada), and stained using the following fluorescence-conjugated antibodies: anti-CD3-PE (clone UCHT1; BioLegend, USA), anti-CD4-FITC (clone PRA-T4; BioLegend, USA), anti-CD45RA-PE-Cy7 (clone HI100; BioLegend, USA), and pSTAT3-Alexa Fluor 647 (clone 13A3-1, BioLegend, USA). Samples were then analyzed by flow cytometry, gating on CD3^+^CD4^+^CD45RA^+^ based on fluorescent minus one staining controls. Flow cytometry experiments were carried out on 4-laser LSR II or Fortessa flow cytometers (BD Biosciences, USA). Data were analyzed using FlowJo v10 (Ashland, USA).

For SMAD2/3 phosphorylation, negatively-depleted naïve CD4 T cells were serum starved overnight in RPMI at a density of 2 million cells per ml. The next day, cells were stimulated with recombinant TGF-β (R&D systems; cat# 240-B-002/CF) at a final concentration of 20 ng/ml for one hour and then fixed using pre-warmed Cytofix^TM^ buffer (BD Biosciences, Canada), permeabilized using Perm III buffer (BD Biosciences, Canada), and stained using the following fluorescence-conjugated antibodies: anti-CD4-FITC (clone PRA-T4; BioLegend, USA), anti-CD45RA-PE-Cy7 (clone HI100; BioLegend, USA), and anti-Smad2 (pS465/pS467)/Smad3 (pS423/pS425)-PE (clone 072-670; BD Biosciences, Canada). Samples were then analyzed by flow cytometry, gating on CD4^+^CD45RA^+^ based on fluorescent minus one staining controls.

# **Western blot**: Magnetic bead-purified neonatal and adult naïve CD4 T cells were washed with PBS and then lysed in RIPA buffer supplemented with phosphatase/protease inhibitors (Santa Cruz Biotechnology, #sc-24948). Measurement of protein concentration was performed using Pierce 660 nm protein assay (Thermo Fisher Scientific, #22662). Lysates were boiled in 4X Laemmeli buffer (Bio-Rad) supplemented with 2-mercaptoethanol, ran on a 4-20% Tris-Glycine mini protein gradient gel (Thermo Fisher Scientific, #XP04202BOX) and transferred to a PVDF membrane (Bio-Rad, 1620177). The membrane was incubated with β-Actin (8H10D10) mouse mAb (Cell Signaling Technology) and Smad2/3 (D7G7) XP^®^ rabbit mAb (Cell Signaling Technology) as primary antibodies and IRDye^®^ 680RD Goat anti-Mouse IgG (LI-COR) and IRDye^®^ 800CW Donkey anti-Rabbit IgG (LI-COR) as secondary antibodies. The Blot was imaged using a LI-COR Odyssey 9120 machine and image was processed using Image Studio Lite ver. 5.2 software.

**RNA extraction and gene expression profiling:** Total RNA was extracted from frozen (-80 ºC) FACS-isolated T cells pellet in one batch after treating the samples using QIA shredder columns and the RNeasy Mini Kit (Qiagen). To ensure purity, RNA samples were cleaned using RNA Clean & Concentrator kit (Zymo Research Corporation, USA). Sample RNA quantities were measured with a NanoDrop spectrophotometer (Thermo Fisher Scientific, USA). Prior to loading on bead chip arrays, sample integrity was evaluated using Agilent RNA 6000 Nano kit and Agilent 2100 Bioanalyzer (both Agilent, USA). All samples yielded RNA Integrity Numbers (RIN) greater than 9.3. RNA samples were then hybridized to the Illumina HumanHT-12_v4_BeadChip array. The resulting data were transferred to GenomeStudio (Illumina); R was then used to further process and normalize the data, using the lumi^[1](#_ENREF_1)^ and limma^[2](#_ENREF_2)^ packages, after filtering out of low signal intensity (with signal intensity<100) and hemoglobin gene probes[^3^](#_ENREF_3). We used false discovery rate (FDR) <5% and adjustment for multiple testing was done using the Benjamini & Hochberg method[^4^](#_ENREF_4).

To validate gene array data by RT-PCR, total RNA was converted to cDNA using Maxima first strand cDNA synthesis kit (Thermo Fisher Scientific, USA). Real-time PCR was performed in triplicates using Power SYBR Green (Thermo Fisher Scientific, USA). The reactions were incubated at 95°C for 10 min, followed by 40 cycles of 95°C for 15 s and 62°C for 60 s in a ViiA 7 real-time PCR system (Life Technologies, Canada). The primers used for qPCR confirmation are listed in Supplemental Table S6 with primer efficiencies >92%. Average Ct values were calculated using the 2^-ΔΔCt^ method[^5^](#_ENREF_5).

**Gene Set Enrichment Analysis (GSEA):** Analysis was performed using GSEA software v3.02[^6^](#_ENREF_6)^,^[^7^](#_ENREF_7). Detectably expressed gene probes (n=17,418) were used as input for the GSEA. GSEA was performed separately for the Hallmark, BioCarta, KEGG, Reactome, and Gene Ontology (GO) gene sets; pathways with a false-discovery rate (FDR) <5% (including their leading-edge genes) are presented in Supplemental Table S4. No gene pathways were enriched for genes from KEGG, Reactome, and Gene Ontology at FDR<5%. For graphical gene ontology representation, we used Cytoscape v3.6.03[^8^](#_ENREF_8) and ClueGO plugin v2.3.34[^9^](#_ENREF_9); all 7,137 differentially expressed probes were used as input. For the GO biological processes analysis, functional enrichment was determined by sorting enriched terms based on a p-value <0.05, adjusting for multiple comparisons using the Bonferonni step-down method. GO tree intervals between 3 and 8, and a κ threshold of 0.4 were used.

In order to evaluate the enrichment of the targets of transcription factors, we extracted transcription factors and their targets from the CHEA (199 transcription factors)[^10^](#_ENREF_10), ENCODE (181 transcription factors)[^11^](#_ENREF_11), JASPER (111 transcription factors)[^12^](#_ENREF_12), and TRANSFAC (201 transcription factors)[^13^](#_ENREF_13) databases, and then performed gene set enrichment analysis (GSEA) separately for each database, using above-intensity probes (n=17,418) from the genome-wide expression array data. Transcription factors from the CHEA and TRANSFAC databases that were enriched with FDR<25% are presented in Supplemental Table S5. GSEA using ENCODE and JASPER databases did not yield any transcription factors at FDR<25%.

**STAT3 cloning:**  After extracting total RNA from adult peripheral blood mononuclear cells, the human *STAT3* gene was amplified via RT-PCR using *STAT3* forward (5′-TGCTAGCAGGATGGCCCAATGGAATCAG-3′) and reverse (5′-TGAGCTCTCACATGGGGGAGGTAGCGC-3′) primers. The gene was then cloned into a pIRES2-eGFP plasmid (<http://www.dmlim.net/vectors/pIRES2-EGFP/pIRES2-EGFP-map.pdf>). Integrity of the plasmid insert with flanking regions was confirmed by Sanger sequencing.

**Supplemental references**

1. Du P, Kibbe WA, Lin SM. lumi: a pipeline for processing Illumina microarray. Bioinformatics 2008; 24:1547-8.

2. Ritchie ME, Phipson B, Wu D, Hu Y, Law CW, Shi W, et al. limma powers differential expression analyses for RNA-sequencing and microarray studies. Nucleic Acids Res 2015; 43:e47.

3. de Goede OM, Razzaghian HR, Price EM, Jones MJ, Kobor MS, Robinson WP, et al. Nucleated red blood cells impact DNA methylation and expression analyses of cord blood hematopoietic cells. Clin Epigenetics 2015; 7:95.

4. Benjamini Y, Hochberg Y. Controlling the False Discovery Rate: A Practical and Powerful Approach to Multiple Testing. Journal of the Royal Statistical Society 1995; 57:289-300.

5. Livak KJ, Schmittgen TD. Analysis of relative gene expression data using real-time quantitative PCR and the 2(-Delta Delta C(T)) Method. Methods 2001; 25:402-8.

6. Subramanian A, Tamayo P, Mootha VK, Mukherjee S, Ebert BL, Gillette MA, et al. Gene set enrichment analysis: a knowledge-based approach for interpreting genome-wide expression profiles. Proc Natl Acad Sci U S A 2005; 102:15545-50.

7. Mootha VK, Lindgren CM, Eriksson KF, Subramanian A, Sihag S, Lehar J, et al. PGC-1alpha-responsive genes involved in oxidative phosphorylation are coordinately downregulated in human diabetes. Nat Genet 2003; 34:267-73.

8. Shannon P, Markiel A, Ozier O, Baliga NS, Wang JT, Ramage D, et al. Cytoscape: a software environment for integrated models of biomolecular interaction networks. Genome Res 2003; 13:2498-504.

9. Bindea G, Mlecnik B, Hackl H, Charoentong P, Tosolini M, Kirilovsky A, et al. ClueGO: a Cytoscape plug-in to decipher functionally grouped gene ontology and pathway annotation networks. Bioinformatics 2009; 25:1091-3.

10. Lachmann A, Xu H, Krishnan J, Berger SI, Mazloom AR, Ma'ayan A. ChEA: transcription factor regulation inferred from integrating genome-wide ChIP-X experiments. Bioinformatics 2010; 26:2438-44.

11. Consortium EP. An integrated encyclopedia of DNA elements in the human genome. Nature 2012; 489:57-74.

12. Fornes O, Castro-Mondragon JA, Khan A, van der Lee R, Zhang X, Richmond PA, et al. JASPAR 2020: update of the open-access database of transcription factor binding profiles. Nucleic Acids Res 2020; 48:D87-D92.

13. Wingender E. The TRANSFAC project as an example of framework technology that supports the analysis of genomic regulation. Brief Bioinform 2008; 9:326-32.
